# Antimicrobial Resistance in Wildlife in Guadeloupe (French West Indies): Distribution of a Single *bla*_CTX–M–1_/IncI1/ST3 Plasmid Among Humans and Wild Animals

**DOI:** 10.3389/fmicb.2020.01524

**Published:** 2020-07-10

**Authors:** Stephanie Guyomard-Rabenirina, Yann Reynaud, Matthieu Pot, Emmanuel Albina, David Couvin, Celia Ducat, Gaëlle Gruel, Severine Ferdinand, Pierre Legreneur, Simon Le Hello, Edith Malpote, Syndia Sadikalay, Antoine Talarmin, Sebastien Breurec

**Affiliations:** ^1^Transmission, Reservoir and Diversity of Pathogens Unit, Institut Pasteur de la Guadeloupe, Pointe-a-Pitre, France; ^2^UMR ASTRE, CIRAD, Montpellier, France; ^3^UMR ASTRE, F-34398, INRA, CIRAD, Université de Montpellier, Montpellier, France; ^4^Inter-University Laboratory of Human Movement Biology-EA 7424, University of Lyon, University Claude Bernard Lyon 1, Villeurbanne, France; ^5^Enteric Bacterial Pathogens Unit, Institut Pasteur, Paris, France; ^6^UNICAEN, Groupe de Recherche sur l’Adaptation Microbienne, GRAM 2.0, EA2656, University of Caen Normandy, Caen, France; ^7^Laboratory of Clinical Microbiology, University Hospital of Guadeloupe, Pointe-a-Pitre/Les Abymes, France; ^8^Faculty of Medicine Hyacinthe Bastaraud, University of the Antilles, Pointe-a-Pitre, France; ^9^INSERM, Center for Clinical Investigation 1424, Pointe-a-Pitre/Les Abymes, France

**Keywords:** *Escherichia coli*, wild animals, antimicrobial resistance, extended-spectrum beta-lactamase, plasmid

## Abstract

Limited data are available on the contribution of wildlife to the spread of antibacterial resistance. We determined the prevalence of resistance to antibiotics in *Escherichia coli* isolates collected from wild animals in 2013 and 2014 and the genetic basis for resistance to third-generation cephalosporin in Guadeloupe. We recovered 52 antibiotic-resistant (AR) *E. coli* strains from 48 of the 884 (5.4%) wild animals tested (46 iguanas, 181 birds, 289 anoles, and 368 rodents at 163 sampling sites). Rodents had higher rates of carriage (*n* = 38, 10.3%) than reptiles and birds (2.4% and 1.1%, respectively, *p* < 0.001). A significant association (*p* < 0.001) was found between the degree of anthropization and the frequency of AR *E. coli* carriage for all species. The carriage rate of ciprofloxacin- and cefotaxime-resistant isolates was 0.7% (6/884) and 1.5% (13/884), respectively. Most (65.4%) AR *E. coli* were multi-drug resistant, and the prevalence of extended-spectrum beta-lactamase (ESBL)-producing *E. coli* was low (*n* = 7, 0.8%) in all species. Eight ESBL-producing *E. coli* were recovered, two genetically unrelated isolates being found in one bird. These isolates and 20 human invasive ESBL *E. coli* isolates collected in Guadeloupe during the same period were investigated by whole genome sequencing. *bla*_CTX–M–1_ was the only ESBL gene shared by three animal classes (humans, *n* = 2; birds, *n* = 2; rodents, *n* = 2). The *bla*_CTX–M–1_ gene and most of the antimicrobial resistance genes were present in a large conjugative IncI1 plasmid that was highly similar (>99% nucleotide identity) to ESBL-carrying plasmids found in several countries in Europe and in Australia. Although the prevalence of ESBL-producing *E. coli* isolates was very low in wild animals, it is of concern that the well-conserved IncI1 plasmid-carrying *bla*_CTX–M–1_ is widespread and occurs in various *E. coli* strains from animals and humans.

## Introduction

Antimicrobial resistance (AMR) has become a major threat for human health worldwide, resulting in dramatic increases in morbidity and mortality (Laxminarayan et al., 2013). AMR is a complex, multifaceted problem involving humans, animals, and the environment; however, the role of wildlife in the spread of antibacterial resistance might be underestimated. The occurrence of antibiotic-resistant (AR) bacteria in wild fauna has been reported increasingly in diverse animal species in a wide range of habitats and locations since the first description in 1977 of chloramphenicol-resistant *Escherichia coli* isolates in Japanese wild birds (Sato et al., 1978; Wang et al., 2017).

Wild fauna, however, may not be exposed to antibiotics and should be protected from AR bacteria in view of their possible role in their transmission. The environment receives antibiotic residues and AR bacteria in human and animal waste, resulting in indirect exposure of wild fauna. Third-generation cephalosporin-resistant (3GC-R) Enterobacteriaceae have spread throughout the world, initially in hospitals and more recently in communities. Resistance is mediated mainly by acquired extended-spectrum beta-lactamase (ESBL) genes located on mobile genetic elements. ESBL enzymes can hydrolyze almost all beta-lactams (except for carbapenems and cephamycins) and are frequently associated with genes resistant to many types of antimicrobial agent (Peirano and Pitout, 2019). Since the first report of ESBL-producing *E. coli* isolates from wild animals in Portugal in 2006, at least 80 wildlife species worldwide have been reported to carry ESBL-producing Enterobacteriaceae (Wang et al., 2017). They have been reported mostly in waterfowl, birds of prey, and rats (Pinto et al., 2010; Guenther et al., 2012, 2013; Veldman et al., 2013) but also in species living in environments that are less exposed to human activities (Guenther et al., 2012). Most ESBL-producing bacterial pathogens in wild animals are *E. coli*, because of its ubiquity and its ability to acquire the AR gene through mobile genetic elements.

Guadeloupe, a French overseas territory located in the Caribbean, was considered a very high-resource country in 2013^1^. One-third of the small island is devoted to agriculture, and the mountains are sparsely populated, resulting in a very high population density (247.7 inhabitants/km^2^ in 2013). The diversity and population of wild fauna are limited because of extensive hunting during the colonization period (Grouard, 2007; Lorvelec et al., 2007), and only a few animal classes have been described: mammals (agouti, mongooses, raccoons, bats, rats, and mice), reptiles and amphibians (frogs, toads, iguanas, anoles, geckos and turtles), and birds (278 listed species, including a number of migratory species)^2^ (Powell and Henderson, 2005; Davalos and Turvey, 2012). Most of the wild fauna live in close proximity to humans, and small reptiles such as anoles and geckos live inside dwellings (Powell and Henderson, 2005). Rats and mice proliferate on the island because of poor waste management and crops (fruit trees, sugar cane) that favor their increase. Birds such as bananaquit are also found around dwellings.

Few data on AMR in Guadeloupe are available. Although Enterobacteriaceae carbapenemase-producing strains have emerged (Bastian et al., 2015; Breurec et al., 2015), studies on community-acquired urinary tract infections (Guyomard-Rabenirina et al., 2016) and waste water treatment plants (Guyomard-Rabenirina et al., 2017) showed a low prevalence of ESBL-producing Enterobacteriaceae. Previous studies suggested that wildlife, particularly terrestrial, could be used as sentinels of environmental AMR (Furness et al., 2017). The primary objective of our study was to determine the prevalence of AR *E. coli* in the feces of wild animals. The secondary objectives were to determine the influence of human activities on the spread of AR *E. coli* in wild fauna, to use whole genome sequencing to identify the genetic background of ESBL-producing *E. coli* collected from wild animals and the genetic basis for resistance to 3GC, and to compare the genomic features of isolates with those from humans collected during the same period.

## Materials and Methods

### Sampling

**FIGURE 1:**
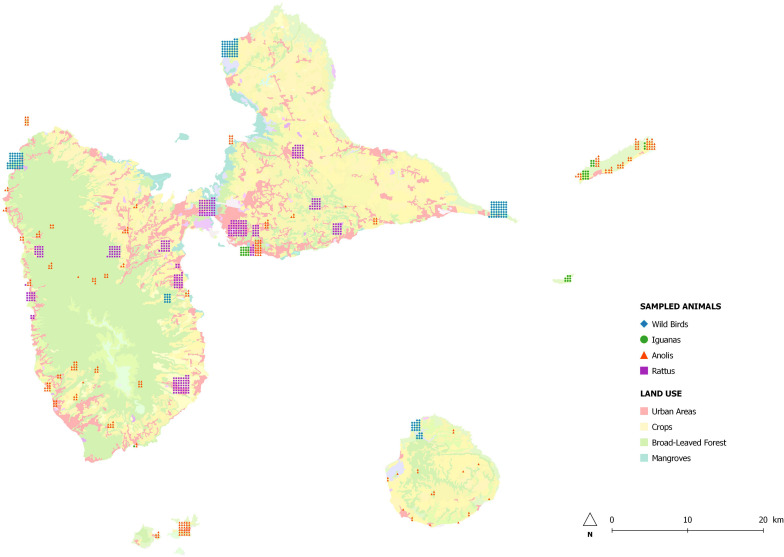
Map of Guadeloupe and location of sampling sites.

We sampled 884 animals between February 2013 and February 2014 at sites throughout Guadeloupe and nearby islands (Les Saintes, Marie-Galante, La Désirade, and Petite-Terre) identified with the geographical information system Q-GIS (Figure 1). A single cloacal swab was taken from 181 birds at 46 sampling sites and from 289 anoles at 76 sampling sites. Feces from 46 iguanas living in colonies were collected at six sampling sites. As part of an ancillary study, fragments of colon were collected during dissection of 368 rodents trapped live at 35 sampling sites and placed in sterile water. Species were identified according to morphological criteria.

All the procedures were approved by the regional environment, planning, and housing agencies and by the Guadeloupe National Park. The project was also approved by the Committee for Ethics in animal experiments of the French West Indies and Guyana (references 69-2012-4; 69-2012-6; 69-2012-7). Animals were cared for and used according to the French decree No. 2013-118 of February 1, 2013, on the protection of animals, which meets European Union Directive 2010/63 on the protection of animals used for experimental and other scientific purposes.

In order to investigate the association between carriage of AR bacteria and proximity to human activities, sampling sites were classified into two groups according to their degree of anthropization with Q-GIS: (i) wilderness with no human presence or countryside with limited human activities, and (ii) human-perturbed landscapes with a matrix of agriculture and livestock activities, and urban and suburban areas with high levels of human activity.

### *E. coli* Isolation and Antimicrobial Susceptibility Analysis

Buffered peptone water was added to the samples, which were then shaken manually. After incubation overnight at 37°C, 100 μL of the suspension was inoculated onto three lactose-TTC-agars supplemented with Tergitol-7, each containing a different antibiotic: 2 mg/L of ampicillin, 2 mg/L of cefotaxime, or 1 mg/L of ciprofloxacin. They were incubated at 37°C for 24 h (SFM and EUCAST, 2014). Presumptive *Enterobacteriaceae* colonies on lactose-TTC-agar (orange colonies, oxidase-negative, Gram-negative bacilli) were isolated randomly and identified by matrix-assisted laser-desorption/ionization time-of-flight mass spectrometry on an Axima Performance (Shimadzu Corp., Osaka, Japan). Colony morphology was investigated as a means of screening isolates with different antibiotic susceptibilities. Three colonies were identified randomly for each identical morphology.

Susceptibility to amoxicillin (10 μg), amoxicillin/clavulanic acid (20 μg/10 μg), ticarcillin (75 μg), cephalothin (30 μg), cefotaxime (30 μg), ceftazidime (30 μg), cefoxitin (30 μg), aztreonam (30 μg), imipenem (10 μg), gentamicin (15 μg), amikacin (30 μg), trimethoprim/sulfamethoxazole (1.25/23.75 μg), nalidixic acid (30 μg), ciprofloxacin (5 μg), and tetracycline (30 UI) was tested by the disk diffusion method on Mueller–Hinton agar (Bio-Rad, Marnes-la-Coquette, France), and production of ESBL was detected by the double-disk synergy test, according to the 2014 guidelines of CA-SFM/EUCAST (SFM and EUCAST, 2014). Isolates depicting resistant or intermediate phenotype were classified together for analysis. Growth inhibition diameters were measured with the Adagio automated system (Bio-Rad). *E. coli* ATCC 25922 was used as the control strain. If more than one *E. coli* strain with the same antibiotic susceptibility pattern was isolated from the same animal, only one randomly chosen isolate was analyzed. An AR *E. coli* isolate was defined as a strain resistant to at least one of the antibiotics tested and a multi-drug-resistant *E. coli* as an isolate resistant to three or more antimicrobial classes (Magiorakos et al., 2012).

### Core Genome Phylogenetic Analyses and Antibiotic Resistance Genes

Whole genome sequencing was performed at the “Plateforme de microbiologie mutualisée” of the Pasteur International Bioresources Network (Institut Pasteur, Paris, France) on eight ESBL-producing *E. coli* isolates collected from wild animals for this study and on all human ESBL-producing *E. coli* isolates (*n* = 20) recovered during the same period from patients admitted to the University Hospital of Guadeloupe for community-acquired (*n* = 4) and nosocomial (*n* = 16) bloodstream infections. Isolates were considered to be community-acquired if recovered by culture from a sample obtained within 48 h after admission. The study protocol was approved by the Ethics Committee of the University Hospital of Guadeloupe (reference A5_19_12_05_TRAMID).

DNA was extracted with a DNA Mini Kit (Qiagen). The libraries were prepared with a Nextera XT Kit (Illumina), and sequencing was performed with the NextSeq 500 system (Illumina), generating 100–151 base-pair (bp) end reads, which have been deposited in the NCBI-SRA public archives under the project’s accession number PRJNA600948. Reads were trimmed and filtered with an AlienTrimmer (Criscuolo and Brisse, 2014) and a quality Phred score threshold of 13 on a minimum length of 30 nucleotides, yielding a mean of 98-fold estimated coverage (minimum, 82-fold; maximum, 149-fold). Reads were then assembled *de novo* with SPAdes v3.9.0 (Bankevich et al., 2012) and the “–careful” option. The quality of assemblies was assured with QUAST software (Gurevich et al., 2013), resulting in a mean N50 of 81 916 (minimum, 48 588; maximum, 173 865). The assembled genomes were annotated with Prokka (Seemann, 2014). A core genome was extracted with Roary software (Page et al., 2015). Recombination sequences were identified and removed from the global core genome alignment with fastGEAR software (Mostowy et al., 2017), giving a global alignment of 1 784 574 bp, 3082 genes shared by 100% of bacterial isolates and 47 690 polymorphic sites. Maximum likelihood (ML) phylogenetic reconstruction was performed with RAxML v8 (Stamatakis, 2014), the GTR-CAT model, and 100 bootstrap replicates, and the tree was drawn with iTOL (Letunic and Bork, 2019). The content of antibiotic resistance genes was assessed with ResFinder (Zankari et al., 2012), and plasmids were identified with PlasmidFinder (Carattoli et al., 2014).

Previously described polymerase chain reaction (PCR) methods were used to screen for genes encoding plasmid-encoded *bla*_CTX–M_, *bla*_TEM_, and *bla*_SHV_ beta-lactamase genes (Guyomard-Rabenirina et al., 2016).

### Plasmid Sequencing by Oxford Nanopore Technology and Syntenic Analysis

PlasmidFinder and ResFinder were used to identify an IncI1/sequence type (ST3) signature and the presence of an ESBL gene *bla*_CTX–M–1_ in six bacterial strains (EC1, EC7, EC23, EC28, EC38, and EC45). Such profile has been described in a previous IncI1/*bla*_CTX–M–1_/ST3 assembled and annotated plasmid called pESBL20150178 (GenBank accession number MK181568) purified from a strain of *E. coli* sampled from a bloodstream infection in Denmark in 2015 (Valcek et al., 2019). In order to fully sequence plasmids in EC1, EC7, EC28, and EC38 isolates, we performed a nanopore sequencing [Oxford Nanopore Technology^®^ (ONT))]^3^. Libraries were prepared following manufacturer instructions using the SQK-LSK109 Ligation Sequencing Kit with the EXP-NBD104 Native Barcoding Expansion 1–12 Kit and performed without optional shearing steps to select for long reads. FLO-MIN106 flowcell connected to the MinION device were used to sequence the library during 48 h using the MinKNOW software (ONT). MinION reads were base-called using Guppy software v3.6.0 and further de-barcoded and screened for quality (resulting in mean *q* > 11) using EPI2ME v2020.2.10 “Fastq Barcoding worklow.” We obtained a mean reads size of 6343 bp (minimum, 4405; maximum, 8541) and a mean of 156-fold estimated coverage (minimum, 124-fold; maximum, 193-fold). A hybrid assembly was then performed using both high-quality Illumina and nanopore reads thanks to Unicycler pipeline (Wick et al., 2017). PlasmidFinder and pMLST were used to correctly identify contigs corresponding to IncI1/ST3 plasmids in each assembly. Using this approach we successfully recovered the full sequence of a circularized plasmid in strains EC1, EC7, EC28, and EC28, so-called pEC1, pEC7, pEC28, and pEC38. Plasmids sequences have been deposited in the GenBank database (accession numbers pEC1: CP053560;pEC7: CP053679; pEC28: CP053678; and pEC38: CP053677). Antibiotic resistances were further confirmed by ResFinder. A syntenic analysis of all plasmids (including pESBL20150178) versus pEC1 was generated with BRIG software (Alikhan et al., 2011). The reference plasmid pEC1 was annotated using RAST (Aziz et al., 2008). The nucleotide sequence of pESBL20150178 was searched with Mash Screen (Ondov et al., 2019) in the PLSDB database (a resource containing 16 168 plasmid sequences collected from the NCBI nucleotide database) (Galata et al., 2018). All plasmids were further aligned using Mauve software (Darling et al., 2004).

### Statistical Analysis

Microsoft Access 2003 was used for data entry and Stata Version 10 for statistical analysis. In univariate analyses, the χ^2^ test (or Fisher’s exact test when appropriate) was used to compare categorical data. We considered *p*-values < 0.05 to be significant.

## Results

### Prevalence of Antibiotic-Resistant *E. coli*

A total of 52 AR *E. coli* isolates were recovered from 48 (5.4%) of the 884 wild animals tested (46 iguanas, 181 birds, 289 anoles, and 368 rodents at 163 sampling sites). Rodents had a higher rate of carriage (*n* = 38, 10.3%) than reptiles and birds (2.4% and 1.1%, respectively, *p* < 0.001). Among rats, the frequency of carriage was significantly higher in *Rattus norvegicus* (*n* = 23, 14.2%) than *Rattus rattus* (*n* = 12, 6.4%) (*p* < 0.05). Among reptiles, only the invasive species of iguana (*Iguana iguana*) and the most common species of anoles (*Anolis marmoratus*) in Guadeloupe were carriers of AR *E. coli* (Table 1).

**TABLE 1:** Wild animal species sampled and the prevalence of antibiotic resistant-, multidrug resistant-, and extended spectrum beta-lactamase producing-*Escherichia coli* from respective host species from Guadeloupe (French West Indies).

Animals		Number (*N*)	*E. coli*			Sampling sites (*N*)
Classes	Species		AR^a^*N* (%)	MDR^b^*N* (%)	ESB^c^*N* (%)	
Reptiles	*Anolis marmoratus*	234	5 (2.1)	2 (0.8)	0	56
	*Anolis terraealtae*	35	0	0	0	7
	*Iguana delicatissima*	29	0	0	0	4
	*Anolis ferreus*	20	0	0	0	13
	*Iguana iguana*	17	3 (17.6)^d^	3 (17.6)^d^	1 (5.8)	2
Rodents	*Rattus rattus*	187	12 (6.4)^d^	11 5 (5.9)	0	14
	*Rattus norvegicus*	162	23 (14.2)^d^	12 (7.4)	5 (3.1)	15
	*Mus musculus*	19	3 (15.8)	2 (10.5)	0	6
Birds	Bananaquit (*Coereba flaveola*)	60	0	0	0	4
	Lesser Antillean Bullfinch (*Loxigilla noctis*)	27	0	0	0	5
	Yellow Warbler (*Setophaga petechia*)	16	0	0	0	4
	Plumbeous Warbler (*Setophaga plumbea*)	6	0	0	0	2
	Northern Waterthrush (*Parkesia noveboracensis*)	1	0	0	0	1
	American Redstar (*Setophaga ruticilla*)	2	0	0	0	2
	Black-faced Grassquit (*Tiaris bicolor*)	20	0	0	0	4
	Black and White Warbler (*Mniotilta varia*)	1	0	0	0	1
	Blackpoll Warbler (*Dendroica striata*)	2	0	0	0	1
	Black-whiskered Vireo (*Vireo altiloquus*)	17	1 (5.9)	1 (5.9)	0	6
	Caribbean Elaenia (*Elaenia martinica*)	12	0	0	0	4
	Carib Grackle (*Quiscalus lugubris*)	4	1 (25.0)^d^	1 (25.0)^d^	1 (25.0)^d^	2
	Lesser Antillean Saltator (*Saltator albicollis*)	4	0	0	0	1
	Pearly-eyed Thrasher (*Margarops fuscatus*)	1	0	0	0	1
	Scaly-breasted Thrasher (*Margarops fuscus*)	2	0	0	0	2
	Zenaida Dove (*Zenaida aurita*)	2	0	0	0	2
	Bridled Quail Dove (*Geotrygon mystacea*)	1	0	0	0	1
	Common Ground Dove (*Columbina passerina*)	1	0	0	0	1
	Forest Thrush (*Turdus lherminieri*)	1	0	0	0	1
	Green Heron *(Butorides virescens)*	1	0	0	0	1

*^*a*^AR, antibiotic resistant; ^*b*^MDR, multidrug resistant; ^*c*^ESBL, extended spectrum beta-lactamase; ^*d*^two isolates in one animal.*

The carriage rate of ciprofloxacin-, cefotaxime-, and ampicillin-resistant *E. coli* isolates in wild animals was 0.7% (6/884), 1.5% (13/884), and 4.9% (43/884), respectively. Most of the isolates (34/52, 65.4%) were resistant to at least three classes of antibiotics and were thus classified as multi-drug-resistant. *E. coli* isolates were characterized by high rates of resistance to amoxicillin (*n* = 46, 88.5%), ticarcillin (*n* = 46, 88.5%), cephalothin (*n* = 30, 57.7%), tetracycline (*n* = 29, 55.8%), trimethoprim/sulfamethoxazole (*n* = 21, 40.4%), amoxicillin/clavulanic acid (*n* = 18, 34.6%), aztreonam (*n* = 17, 32.7%), cefotaxime (*n* = 14, 26.9%), and ceftazidime (*n* = 14, 26.9%) and by moderate rates of resistance to nalidixic acid (*n* = 11, 21.1%), cefoxitin (*n* = 9, 17.3%), and ciprofloxacin (*n* = 6, 11.5%). No resistance to imipenem, gentamicin, or amikacin was found. We recovered 34 resistance phenotypes; the two most frequent were to amoxicillin and ticarcillin (*n* = 5) and to amoxicillin, ticarcillin, trimethoprim/sulfamethoxazole, and tetracycline (*n* = 9). Of the 14 3GC-R isolates, 8 (0.9% of all 884 animals) were positive in the double-disk synergy test, indicating the presence of an ESBL gene; 5 were from 5 rats, 2 were from 1 bird, and 1 were from 1 iguana. No ESBL gene was recovered among the six isolates with a negative double-disk synergy test using PCR.

Of the 884 animals sampled, 416 (47.1%) were trapped in areas with no or little human activity (wilderness, mangrove forests, and countryside) and 468 (52.9%) in regions with moderate or high human activity (agriculture and livestock activities and suburban and urban areas). The animal class found at the sampling sites depended on the extent of human activity (Table 2). Rodents and reptiles trapped in areas under anthropic influence were not significantly more likely to be carriers of AR bacteria; however, the prevalence of carriage of AR and multi-drug-resistant *E. coli* was significantly higher in areas with moderate or high human activity (*p* < 0.001) (Table 2). We observed no significant difference in the number of ESBL-producing *E. coli* according to the degree of anthropization (*p* = 0.12), probably because few isolates were recovered.

**TABLE 2:** Antibiotic resistant-, multidrug resistant-, and extended-spectrum beta-lactamase producing-*Escherichia coli* according to the level of anthropization.

Classes	Human activities	Animals sampled(*N*)	*E. coli*					
			Antibiotic resistant*N* (%)	*P*-value	Multidrug resistant*N* (%)	*P*-value	ESBL^a^*N* (%)	*P*-value
Rodents	Absence^b^-limited^c^	22	2 (9.1)	>0.5	1 (4.5)	>0.5	0	–
	Moderate^d^-high^e^	346	36 (10.4)		24 (6.9)		5 (1.4)	
Reptiles	Absence-limited	213	4 (1.9)	0.47	1 (0.5)	0.06	0	–
	Moderate-high	122	4 (3.3)		4 (3.3)		1 (0.8)	
Birds	Absence-limited	181	2 (1.1)	–	2 (1.1)	–	1 (0.6)	–
	Moderate-high	0	0		0		0	
All	Absence-limited	416	8 (1.9)	**<0.001**	4 (0.9)	**<0.001**	1 (0.2)	0.12
	Moderate-high	468	40 (8.5)		28 (5.9)		6 (1.3)	

*^*a*^ESBL, extended spectrum beta-lactamase; ^*b*^wilderness area with no human presence; ^*c*^countryside; ^*d*^landscapes with a matrix of agriculture; ^*e*^livestock activities, urban and suburban area. Significant P-values are in bold.*

### Genetic Background of ESBL-Producing *E. coli* Isolates

**FIGURE 2:**
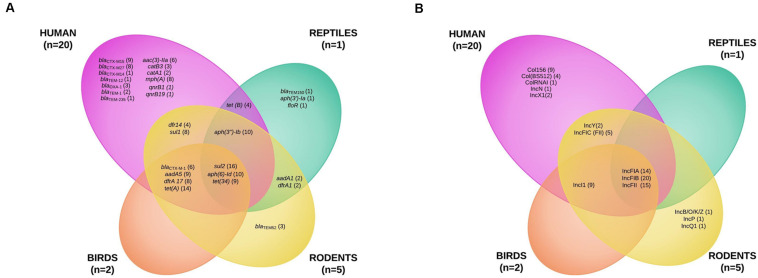
Maximum likelihood (ML) phylogenetic tree of extended-spectrum beta-lactamase-producing *Escherichia coli* isolates based on multiple sequence alignments of the 3082 core genome loci. Sequence type (ST) is indicated for each isolate. Bootstrap values > 60 are indicated on nodes. Hosts and ESBL genes are indicated by vertical colored strips. Other antibiotic resistance genes characterized by ResFinder are indicated by black dots; antimicrobial resistance profiles are represented by triangles: gray for resistance, empty gray for intermediate, and no triangle for susceptibility to corresponding antimicrobial agents.

Maximum likelihood phylogenetic analysis based on 47 690 single nucleotide polymorphisms (SNPs) in the core genomes (3082 genes, global alignment 1784574 bp) of the 28 ESBL-producing *E. coli* revealed clustering into three main branches (Figure 2 and Supplementary Figures S1, S2). The first branch consisted mainly of sequence types (STs) ST131 isolates (*n* = 11) and 1 ST95 and 1 ST1193 isolated from humans; no ST131 *E. coli* was recovered from wild animals. This cluster was the least polymorphic (mean SNP between isolates *n* = 3747, minimum *n* = 3, maximum *n* = 11152). A second branch grouped four clinical samples (ST349, ST38, and 2 ST69) and 1 ST117 isolated from a rat (mean SNP between isolates *n* = 17363, minimum *n* = 751, maximum *n* = 20407). The last branch consisted of six isolates found in wild animals (ST6914, ST1844, 2 ST155, 2 ST196, ST10) and three clinical samples (2 ST410 and ST124) (mean SNP between isolates *n* = 10422, minimum *n* = 2, maximum *n* = 15424). The isolates from humans and wild animals within each cluster were genetically unrelated, and no ST was shared between wild animals and humans.

Of the isolates carrying *bla*_CTX–M–1_, the 6 ESBL-producing *E. coli* isolates belonged to 5 STs: 196, 69, 117, 349, and 124. Two ST196 isolates (EC1 and EC7) from birds and rats trapped at different sites were almost identical, with four core genome multilocus sequence typing allelic mismatches.

### Genetic Analysis of Antimicrobial Resistance Genes and Associated Plasmids

**FIGURE 3:**
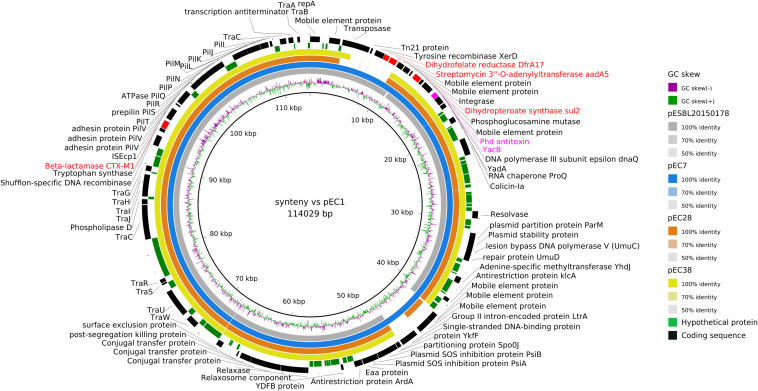
Venn diagram of the antibiotic resistance genes **(A)** and of the plasmid incompatibility groups recovered from extended-spectrum beta-lactamase-producing *Escherichia coli* isolates from humans and wildlife **(B)**.

To better characterize the circulation of AMR genes among ESBL-producing *E. coli* collected from wild animals and humans, 20 human *E. coli* isolates collected from bloodstream infections were randomly selected during the study period and added to the eight isolates recovered from wild animals for whole genome sequencing analysis. We found 30 different resistance genes in the isolates (Figure 3A and Supplementary Tables S1, S2). The distribution varied considerably by animal class, some being present only in the human or wild animal reservoir and others common to both (Figure 3A). The only genes shared by all animal classes and the human isolates conferred resistance to sulfonamides (*sul2*) and tetracycline [*tet(34)*]. Chloramphenicol resistance genes (*catA1* and *catB3*) and quinolone resistance genes *qnr* (*qnrB1* and *qnrB19*) were found only in the human strains (Figure 3A and Supplementary Tables S1, S2).

The *bla*_CTX–M–1_ ESBL encoding gene was the only gene shared by three animal classes (birds, *n* = 2; humans, *n* = 2; rodents, *n* = 2) and not in reptiles. The other *bla*_CTX–M_ genes were found only in human isolates, the most common being *bla*_CTX–M–15_ (9) and *bla*_CTX–M–27_ (8). The other ESBL genes found in wild animals were *bla*_TEM–52_ in three rats and *bla*_TEM–150_ in one iguana (Figure 3A and Supplementary Tables S1, S2). IncF plasmids were the most prevalent in all isolates (23 of the 28 *E. coli* tested, 82.1%) (Figure 3B and Supplementary Tables S1, S2).

**FIGURE 4:**
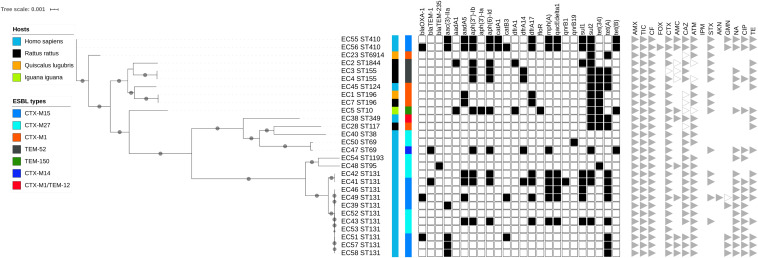
Syntenic analysis of 4 ST3/IncI1/CTX-M1 plasmids versus reference plasmid pESBL20150178. The innermost black ring 1 represents the reference sequence of pEC1. The subsequent rings correspond to GC skew and pairwise comparisons with pESBL20150178, pEC7, pEC28, and pEC38. The last 2 rings represent a genetic map of pEC1: antibiotic resistance genes are indicated by red boxes, and toxin–antitoxin by pink boxes.

As *bla*_CTX–M–1_ strains in our study systematically presented an IncI1/ST3 signature (Supplementary Figure S2), we used ONT sequencing approach for EC1, EC7, EC28, and EC38 *E. coli* isolates to fully sequence plasmids, respectively, named pEC1 (114 029 bp), pEC7 (114 029 bp), pEC28 (113 774 bp), and pEC38 (108 435 bp) (Figure 4). Syntenic analyses revealed that previously described plasmid pESBL20150178 share 95% of its sequence with pEC1 while pEC7 is identical, and pEC28 shares 92% and pEC38 shares 91% with pEC1. Nucleotide identity was >99%. The IncI1 backbone was organized into four major conserved regions encoding replication, stability, leading and conjugative transfer. The *bla*_CTX–M–1_ gene was identified in all plasmidic assemblies, while the *aadA5*, *dfrA17*, and *sul2* genes, conferring resistance to streptomycin, trimethoprim, and sulfonamides, respectively, were characterized only on pEC1 and pEC7. Gene *tet(A)* conferring resistance to tetracyclin was only described in pEC28 and pEC38 (Figure 2). The typical genetic environment of the *bla*_CTX–M–1_ gene was observed, with the tryptophan synthase gene downstream and the ISEcp1 upstream. No gene encoding resistance to heavy-metal ions was detected. A type II toxin–antitoxin system consisting of Phd and YacB was annotated on all plasmids (Figure 4).

During the search for this type of IncI1/*bla*_CTX–M–1_/ST3 plasmid in the PLSDB database, 24 plasmids (from 83 410 to 122 616 bp) sampled only from *E. coli* were identified that shared >99% nucleotide identity with pESBL20150178. All plasmids were defined as IncI1/*bla*_CTX–M–1_/ST3 with the *aadA5*/*dfrA17*/*sul2* region (Supplementary Table S3). All but one (from Australia) plasmid were recovered in isolates from Europe (Denmark, *n* = 10; France, *n* = 4; Netherlands, *n* = 1; Switzerland, *n* = 8). All plasmids except four collected from humans were identified in isolates from livestock.

## Discussion

The overall prevalence of AR *E. coli* in Guadeloupe was low (5.4%), reflecting the low level of resistance observed in *E. coli* from community-acquired urinary tract infections (Guyomard-Rabenirina et al., 2016) and waste water treatment plants (Guyomard-Rabenirina et al., 2017), although the method used may be a partial explanation. We targeted resistance to three antibiotics (ampicillin, cefotaxime, and ciprofloxacin) belonging to two major classes of antibiotics (beta-lactams and fluoroquinolones) used in clinical practice, which may have resulted in underestimation of the level of resistance to other antibiotics such as tetracycline and colistin, which is frequently reported in *E. coli* isolated from wildlife (Shobrak and Abo-Amer, 2014; Ramey et al., 2018; Vogt et al., 2018). The frequency of AR bacteria in wildlife is affected by various factors, which are not yet fully understood, although the anthropogenic impact in areas where they live and feed is a key factor (Bonnedahl and Järhult, 2014; Arnold et al., 2016; Wang et al., 2017; Dolejska and Papagiannitsis, 2018). Our study supports this hypothesis, although no significant difference was observed for any one species, probably because of the few isolates and their inconsistent distribution according to the degree of anthropization. For example, only iguanas belonging to an invasive species and living in close proximity to humans (in a hotel garden and in an urban area) were carriers of AR *E. coli*, as previously described in iguanas in Galapagos (Wheeler et al., 2012).

The carriage rates of AR *E. coli* differed by species. The overall frequency in the birds studied was 1.1%, which is in the lower range of the reported values (1.5–52%) (Radimersky et al., 2010; Hassell et al., 2019) although comparisons are difficult due to differences between the methodologies used. In our study, most of the species sampled were small birds that feed on seeds, fruits, and insects in areas with no or limited human activity. Few data on AMR in reptiles are available, as they are not natural carriers of *E. coli*. Although they are not the best indicator of the distribution of AMR in wildlife, we decided to investigate them because anoles and geckos are considered peridomestic animals, which could be affected by human activity^4^. Despite their close proximity to humans, they had a low rate of carriage (1.7%). Rodents are some of the best studied sentinels of environmental transmission of AMR because of their proximity to human activities. Unsurprisingly, they had the highest rates of carriage of the species included in our study. As previously described (Guenther et al., 2013), *R. norvegicus*, a synanthropic species living in urban and periurban areas, had higher rates of carriage than *R. rattus*, a commensal species closely linked to people and their movements but which readily establishes itself in undeveloped areas, including native forests.

Although most of the AR *E. coli* were multi-drug resistant, ESBL-producing *E. coli* were isolated from only 0.9% of the wild animals and especially rodents (5 of the 8 strains). This frequency is in the lower range of values reported previously for any species (Dolejska and Papagiannitsis, 2018). To the best of our knowledge, we found the first case of ESBL-producing *E. coli* in a wild reptile.

We investigated the genetic relatedness of ESBL-producing *E. coli* from wild animals and humans in Guadeloupe in a “One Health” approach. The *E. coli* from wild animals were not closely related to the isolates that cause human disease in our region, suggesting that wild animals are not a direct source of infection for humans and that human invasive *E. coli* are not passed to wildlife. The pandemic ST131 genetic background that contributed extensively to global dissemination of CTX-M-15 accounted for most human *E. coli* infections in our study; however, it was not reported in wild animals, although it has been found to be dominant in numerous other studies (Wang et al., 2017). ST10, a major clone in humans and in livestock animals, was previously recovered from one iguana (Day et al., 2019; Manges et al., 2019). To the best of our knowledge, only ST117 and ST155 have previously been reported in wild animals (birds) (Dolejska and Papagiannitsis, 2018).

CTX-M-15 and CTX-M-27 were the two dominant ESBL recovered from our human isolates, mainly associated with the ST131 genetic background. CTX-M-15 is currently recognized as the most widely distributed CTX-M beta-lactamase, and CTX-M-27 has begun to emerge worldwide, notably in Europe and Japan (Peirano and Pitout, 2019). Unlike in previous studies (Wang et al., 2017; Dolejska and Papagiannitsis, 2018), we did not find these two variants in our isolates from wild fauna; CTX-M-1 was the only ESBL found in all animal classes except reptiles. In addition to CTX-M enzymes, TEM-52, which has been described in humans, livestock, and wild and companion animals (Saliu et al., 2017), was present in three rats.

The *bla*_CTX–M–1_ ESBL gene from our *E. coli* isolates was present in a large conjugative IncI1 plasmid. The strong similarity in organization, structure, and accessory regions, including the occurrence of a similar AMR gene pattern, indicates a common evolutionary origin. This plasmid was closely similar to the pESBL20150178 plasmid found in a human ESBL-producing *E. coli* isolate in Denmark and also to ESBL-carrying plasmids found in several countries in Europe and in Australia (Supplementary Figure S3), demonstrating its significant role in the worldwide spread of *bla*_CTX–M–1_ genes (Carattoli et al., 2018). Its epidemiological relevance may be even greater, as it has been recovered in *E. coli* isolates from animal and human sources. Its higher frequency in animals than in humans suggests an animal contribution to the CTX-M-1 reservoir in humans through the spread of this specific IncI1/*bla*_CTX–M–1_/ST3 plasmid. Further studies should be conducted in Guadeloupe on the role of livestock and domestic animals in its spread to humans.

To the best of our knowledge, this is the first description of the IncI1/*bla*_CTX–M–1_/ST3 plasmid in the Caribbean islands and of its spread in *E. coli* isolates from humans and wild animals living in the same area. The plasmid was recovered not only in one *E. coli* clone (ST196) in one bird and one rodent but also, more importantly, in *E. coli* with unrelated genetic backgrounds. It has been hypothesized that, under conditions in which a plasmid has a selective advantage, such as resistance to antibiotics or to heavy metals, that increases the probability of survival and/or the growth rates of their host bacteria, the plasmid would become established and the bacteria carrying them would be maintained at a high frequency in the bacterial population (Levin and Stewart, 1980). We did not find any selective advantage of our IncI1/*bla*_CTX–M–1_/ST3 plasmid, except for resistance to antibiotics. Its spread is therefore not favored only by antibiotic selection, as wild animals are unlikely to be directly exposed to clinically relevant antimicrobials. Addiction systems, such as the toxin–antitoxin modules encoded by our plasmid, can prevent loss of a plasmid but cannot compensate for disadvantages imposed by its carriage (Mnif et al., 2010). *In vitro*, however, the fitness cost in the absence of antibiotics imposed on its *E. coli* host by the IncI1/ST3 plasmid carrying the *bla*_CTX–M–1_ gene was negligible (Fischer et al., 2014). This is a concern, as, if this hypothesis is confirmed *in vivo*, the spread of such a gene–plasmid combination in humans and animals will continue despite a reduction in antibiotic use.

Low rates of AR *E. coli* carriage in wild animals in Guadeloupe in 2013 and 2014 were recovered. Although the prevalence of ESBL-producing *E. coli* isolates was very low, our study indicate that well-conserved IncI1/*bla*_CTX–M–1_/ST3 plasmids are spread across wide geographical distances and occur in different *E. coli* strains in animal and human sources.

## Data Availability Statement

The datasets generated for this study can be found in the NCBI with accession number PRJNA600948.

## Ethics Statement

The studies involving human participants were reviewed and approved by the Ethics Committee of the University Hospital of Guadeloupe. The study protocol was approved by the Ethics Committee of the University Hospital of Guadeloupe (reference A5_19_12_05_TRAMID). Written informed consent for participation was not required for this study in accordance with the national legislation and the institutional requirements. The animal study was reviewed and approved by the Committee for Ethics in animal experiments of the French West Indies and Guyana (references 69-2012-4; 69-2012-6; 69-2012-7).

## Author Contributions

SG-R, YR, AT, and SB conceived and designed the study. SG-R, YR, MP, EA, DC, CD, GG, PL, SL, EM, SS, AT, and SB collected biological samples, isolates, and epidemiological data. SG-R, YR, and SB analyzed the data and wrote the manuscript. SF performed the statistical analyses. All authors critically revised the manuscript, read, and approved the final manuscript.

## Conflict of Interest

The authors declare that the research was conducted in the absence of any commercial or financial relationships that could be construed as a potential conflict of interest.

## Footnotes

1 http://hdr.undp.org/

2 https://www.amazona-guadeloupe.com/_iserv/dlfiles/dl.php?ddl=rap-30-2.pdf

3 https://nanoporetech.com/

4 https://www.researchgate.net/publication/312166291_Repartition_des_anoles_de_la_Guadeloupe_I_Basse-Terre_Grande-Terre_ilets_Pigeon_et_ilets_a_Kahouanne

## Supplementary Material

FIGURE S1 Maximum likelihood (ML) unrooted phylogenetic tree of extended-spectrum beta-lactamase-producing *Escherichia coli* isolates based on multiple sequence alignments of the 3082 core genome loci.

FIGURE S2 Maximum likelihood (ML) phylogenetic tree of extended-spectrum beta-lactamase-producing *Escherichia coli* isolates based on multiple sequence alignments of the 3082 core genome loci versus plasmidic incompatibility groups. Sequence type (ST) is indicated for each isolate. Bootstrap values >60 are indicated on nodes. Hosts and ESBL genes are indicated by vertical colored strips. Plasmidic incompatibility groups characterized by plasmid finder are indicated by black dots.

FIGURE S3 Alignment of IncI1/*bla*_CTX–M–1_/ST3 plasmids from the PLSDB database listed in Supplementary Table S3, with the four plasmids sequenced in this study: pEC1, pEC7, pEC28, and pEC38.

TABLE S1 Molecular characteristics of extended-spectrum beta-lactamase-producing *Escherichia coli* isolates from wild animals and humans in Guadeloupe (French West Indies).

TABLE S2 Characteristics of extended-spectrum beta-lactamase-producing *Escherichia coli* isolates from wild animals and humans in Guadeloupe (French West Indies).

TABLE S3 Metadata on IncI1/*bla*_CTX–M–1_/ST3 plasmids in the PLSDB database sharing >99% nucleotide identity with pESBL20150178 and having *aadA5*/*dfrA17*/*sul2* resistance genes.
