# Supplementary figures and images for: Antimicrobial Resistance in Wildlife in Guadeloupe (French West Indies): Distribution of a Single blaCTX–M–1/IncI1/ST3 Plasmid Among Humans and Wild Animals
Source: Front Microbiol. 2020 Jul 10;11:1524. doi: 10.3389/fmicb.2020.01524 (PMC7366356; doi:10.3389/fmicb.2020.01524)

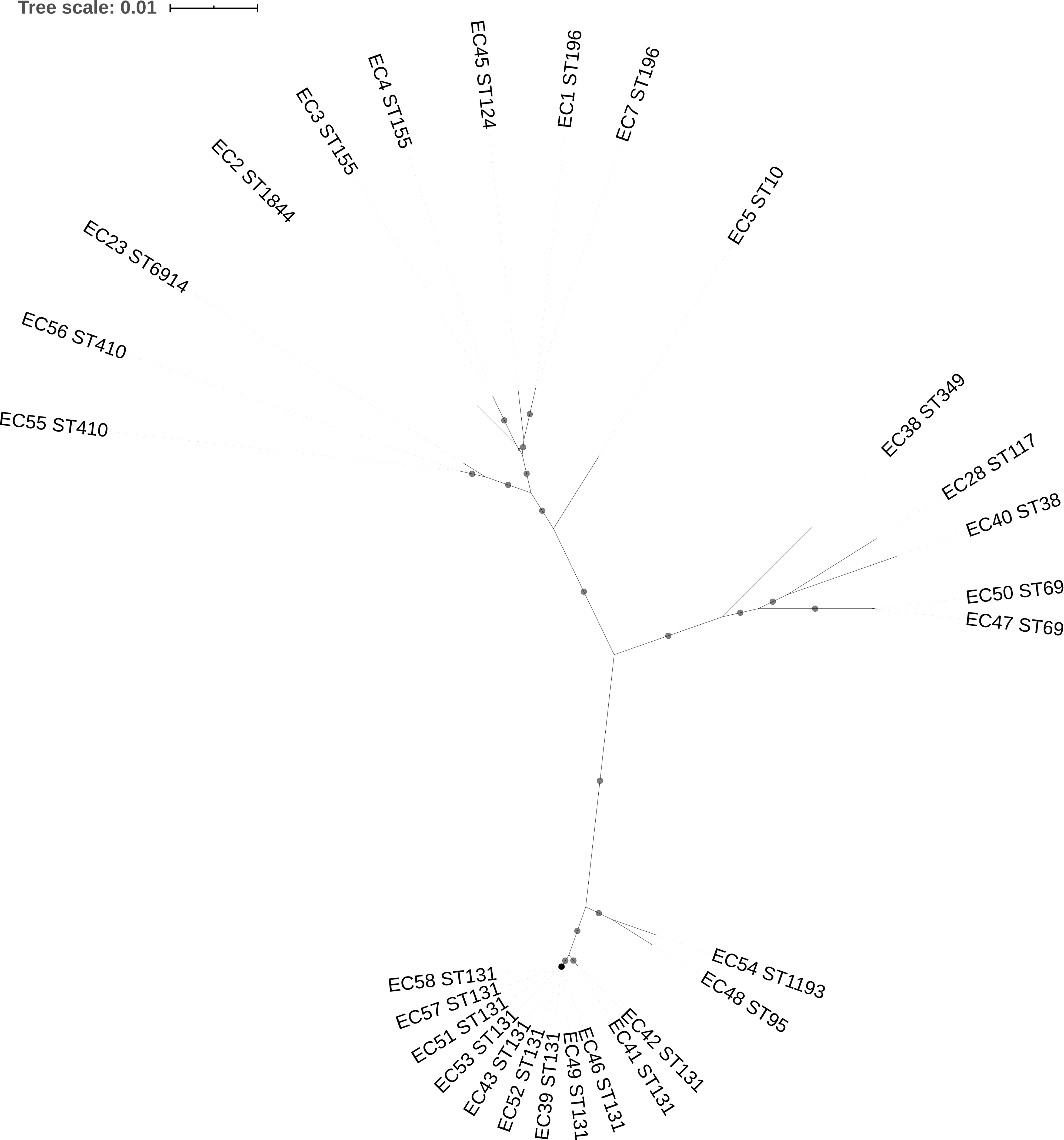

Supplement: FIGURE S1 — Maximum likelihood (ML) unrooted phylogenetic tree of extended-spectrum beta-lactamase-producing Escherichia coli isolates based on multiple sequence alignments of the 3082 core genome loci. [file Image_1.TIFF]

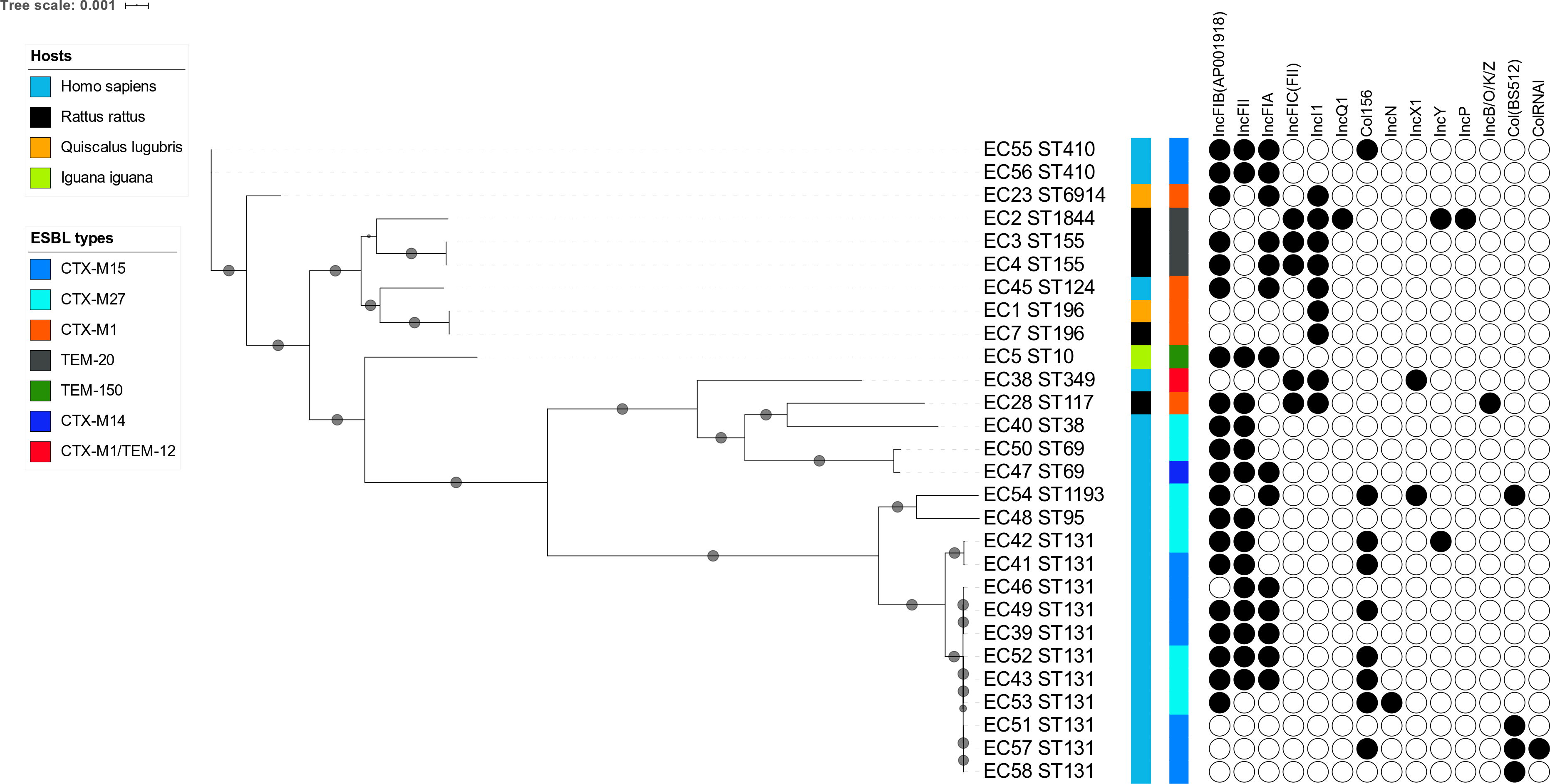

Supplement: FIGURE S2 — Maximum likelihood (ML) phylogenetic tree of extended-spectrum beta-lactamase-producing Escherichia coli isolates based on multiple sequence alignments of the 3082 core genome loci versus plasmidic incompatibility groups. Sequence type (ST) is indicated for each isolate. Bootstrap values >60 are indicated on nodes. Hosts and ESBL genes are indicated by vertical colored strips. Plasmidic incompatibility groups characterized by plasmid finder are indicated by black dots. [file Image_2.TIFF]

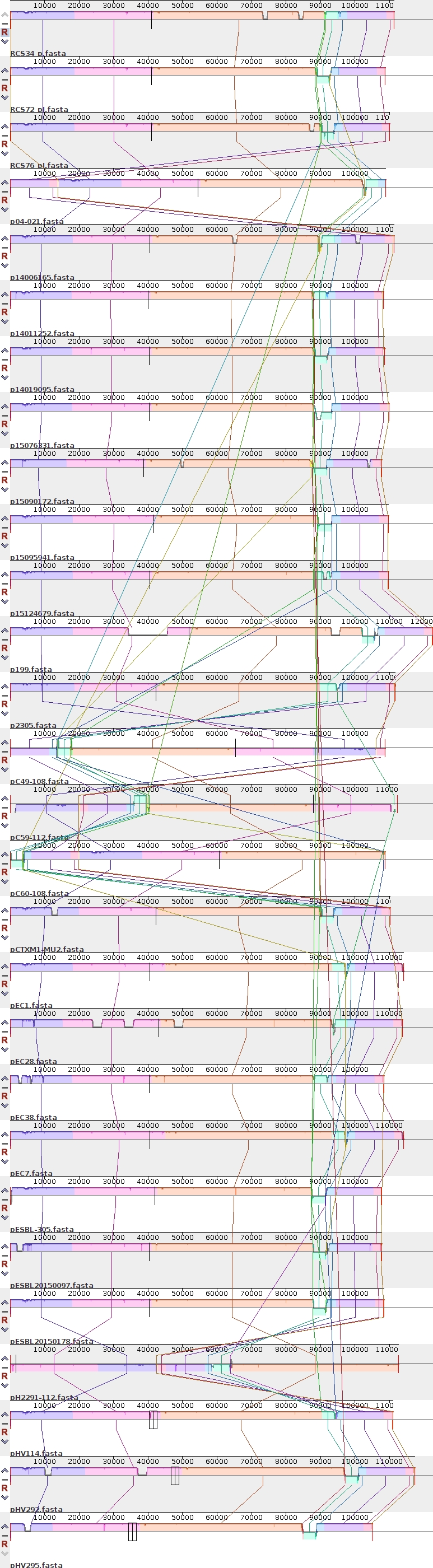

Supplement: FIGURE S3 — Alignment of IncI1/blaCTX–M–1/ST3 plasmids from the PLSDB database listed in Supplementary Table S3, with the four plasmids sequenced in this study: pEC1, pEC7, pEC28, and pEC38. [file Image_3.JPEG]
