# Supplementary material for: Antimicrobial Resistance in Wildlife in Guadeloupe (French West Indies): Distribution of a Single blaCTX–M–1/IncI1/ST3 Plasmid Among Humans and Wild Animals
Source: Front Microbiol. 2020 Jul 10;11:1524. doi: 10.3389/fmicb.2020.01524 (PMC7366356; doi:10.3389/fmicb.2020.01524)
Supplement: TABLE S1 — Molecular characteristics of extended-spectrum beta-lactamase-producing Escherichia coli isolates from wild animals and humans in Guadeloupe (French West Indies). [file Table_1.DOCX]

| **Species** | **Sequence Type** | **ESBL genes** | **Other beta-lactamase genes** | **Other resistance genes** | **Plasmidic incompatibility groups** |
| --- | --- | --- | --- | --- | --- |
| Bird  (2) | 196 (1), 6914 (1) | *bla*_CTX-M1_ (2) | - | *aadA5* (1), *d*frA17 (1), *sul2* (2*), tet(34)* (1), *tet(A*) (1) | IncFIA (1), IncFIB (1), IncI1 (2) |
| Rat  (5) | 117 (1), 155 (2), 196 (1), 1844 (1) | *bla*_CTX-M1_ (2), *bla*_TEM-52_ (3) | - | *aadA1* (1), *aadA5* (1), a*ph(3'')-Ib* (3), *aph(6)-Id* (3), *dfrA1* (1), *dfrA14* (2),  *dfrA17* (1), *sul1* (1), *sul2* (5), *tet(A)* (3), *tet(34)* (4) | IncB/O/K/Z (1), IncFIA (2), IncFIB (3), IncFIC(FII) (4), IncFII (1), IncI1 (5), IncP (1), IncQ1 (1), IncY (1) |
| Iguana  (1) | 10 (1) | *bla*_TEM-150_ (1) | - | *aadA1* (1), *aph(3')-Ia* (1), *aph(3'')-Ib* (1),  *aph(6)-Id* (1), *dfrA1* (1), *floR* (1), *sul2* (1), *tet(34)* (1), *tet(B)* (1) | IncFIB (1), IncFII (1), IncFIA (1) |
| Human  (20) | 38 (1), 69 (2), 95 (1), 124 (1), 131 (11), 349 (1), 410 (2), 1193 (1) | *bla*_CTX-M1_ (2),  *bla*_CTX-M14_ (1), *bla*_CTX-M15_ (9), *bla*_CTX-M27_ (8), *bla*_TEM-12_ (1) | *bla*_OXA-1_ (3), *bla*_TEM-1_ (2), *bla*_TEM-235_ (1) | *aac(3)-IIa (6), aadA5* (7),  *aph(6)-Id* (6), *aph(3'')-Ib* (6),  *catA1* (2), *catB3* (3), *dfrA14* (2), *dfrA17* (6), *mph(A)* (8), s*ul1* (7), *sul2* (8), *qnrB1* (1), *qnrB19* (1), *tet(A)* (10), *tet(B)* (3), *tet(34)* (3) | Col156 (9), Col(BS512) (4), ColRNAI (1), IncFIA (10), IncFIB (15), IncFIC(FII) (1), IncFII (13), IncI1 (2), IncN (1), IncY (1), IncX1 (2) |
| All | 10 (1), 38 (1), 69 (2), 95 (1), 117 (1), 124 (1), 131 (11), 155 (2), 196 (2), 349 (1), 410 (2), 1193 (1), 1844 (1), 6914 (1) | *bla*_CTX-M15_ (9), *bla*_CTX-M27_ (8), *bla*_CTX-M1_ (6),  *bla*_TEM-52_ (3), *bla*_CTX-M14_ (1), *bla*_TEM-12_ (1),  *bla*_TEM-150_ (1) | *bla*_OXA-1_ (3), *bla*_TEM-1_ (2), *bla*_TEM-235_ (1) | *aac(3)-IIa (6), aadA1* (2), *aadA5* (9), *aph(6)-Id* (10),  *aph(3')-Ia* (1), *aph(3'')-Ib* (10),  *catA1* (2), *catB3* (3),  *dfrA1* (2), *dfrA14* (4), *dfrA17* (8),  *floR* (1), *mph(A)* (8), s*ul1* (8), *sul2* (16), *qnrB1* (1), *qnrB19* (1), *tet(A)* (14), *tet(B)* (4), *tet(34)* (9) | Col156 (9), Col(BS512) (4), ColRNAI (1), IncB/O/K/Z (1), IncFIA (14), IncFIB (20), IncFIC(FII) (5), IncFII (15), IncI1 (9), IncN (1), IncP (1), IncQ1 (1), IncY (2), IncX1 (2) |

**Table S1. Molecular characteristics of extended-spectrum beta-lactamases *Escherichia coli* isolates collected in wild animals and humans in Guadeloupe (French West Indies)**
